# Supplementary material for: xia2.multiplex: a multi-crystal data-analysis pipeline
Source: Acta Crystallogr D Struct Biol. 2022 May 18;78(Pt 6):752–69. doi: 10.1107/S2059798322004399 (PMC9159281; doi:10.1107/S2059798322004399)
Supplement: Supplementary file 1 [file d-78-00752-sup1.pdf]

Acta Cryst D
STRUCTURAL
BIOLOGY

Volume 78 (2022)

Supporting information for article:

xia2.multiplex: a multi-crystal data-analysis pipeline

Richard J. Gildea, James Beilsten-Edmands, Danny Axford, Sam Horrell, Pierre Aller, James Sandy, Juan Sanchez-Weatherby, C. David Owen, Petra Lukacik, Claire Strain-Damerell, Robin L. Owen, Martin A. Walsh and Graeme Winter

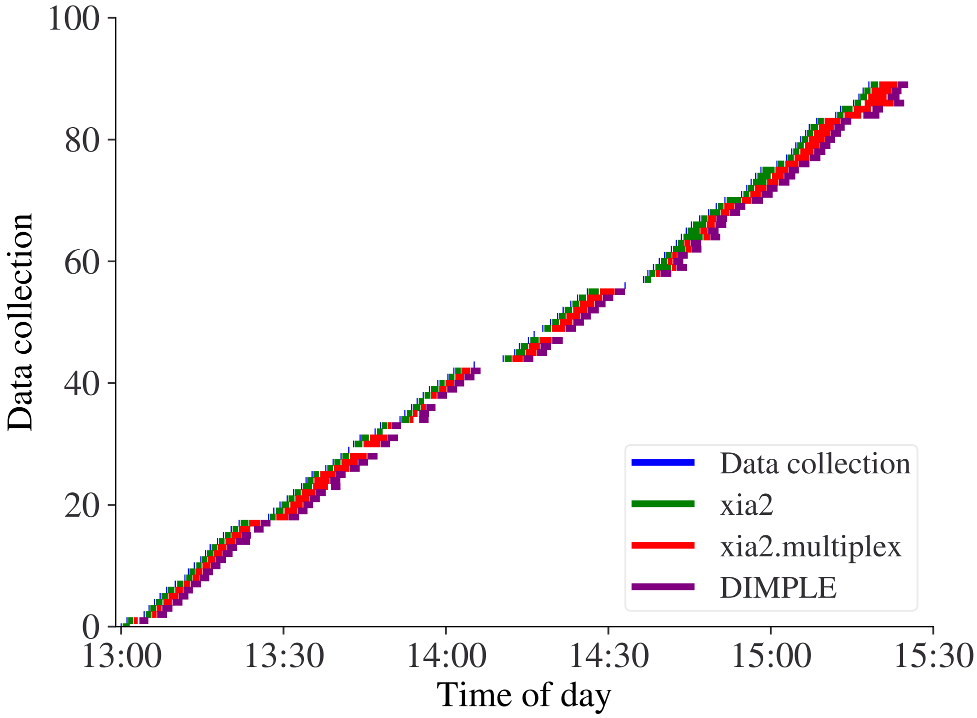

Figure S1: Real-time multi-crystal autoprocessing of a subset of the SARS-CoV-2 main protease data collections reported in §5, using *xia2*, *DIALS*, `xia2.multiplex` and *DIMPLE*. 410 data sets were collected in a single visit at a maximum throughput of 46 data sets per hour. The median time from end of data collection to the completion of the associated processing job was 222.5 s and 352 s for `xia2.multiplex` and *DIMPLE* respectively. 98% of *DIMPLE* results were reported within 10 minutes of data collection finishing.

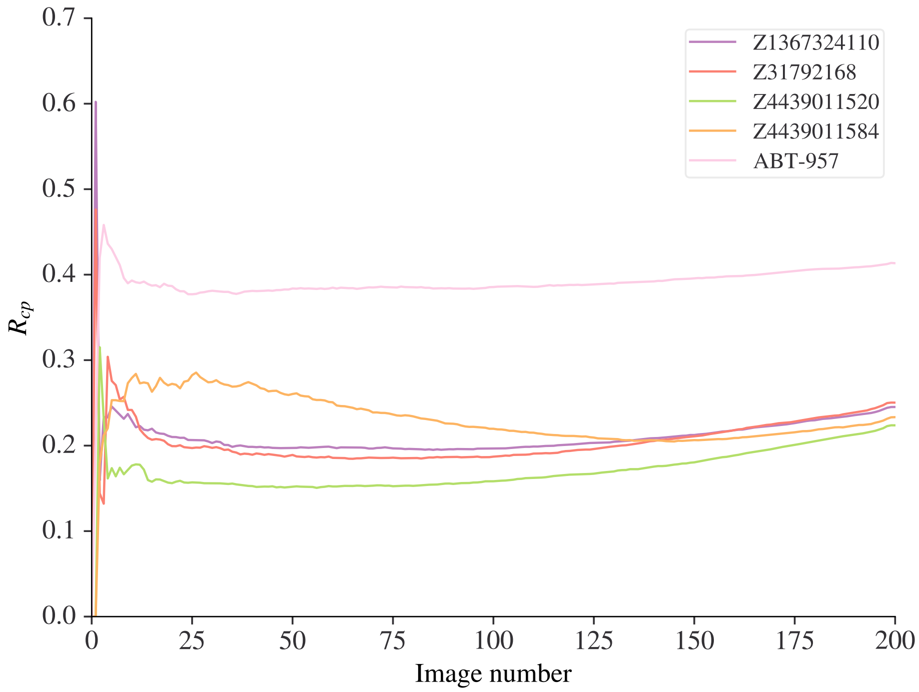

Figure S2: R_{cp} vs image number for the SARS-CoV-2 main protease data collections reported in §5. This suggests some signs of slight radiation damage after around 100 images for the Z1367324110, Z31792168 and Z4439011520 data sets.

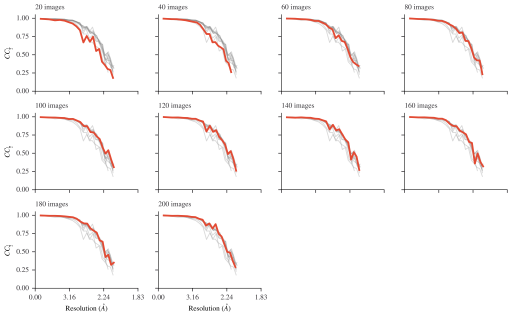

(a)

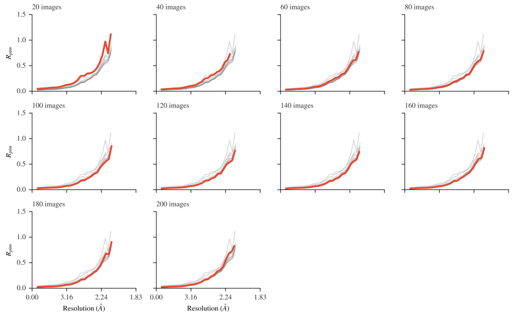

(b)

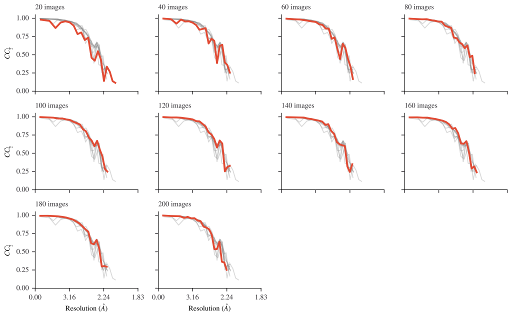

(c)

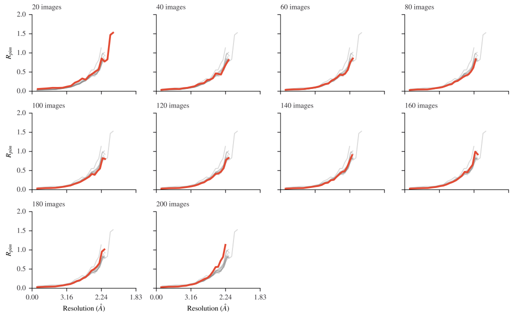

(d)

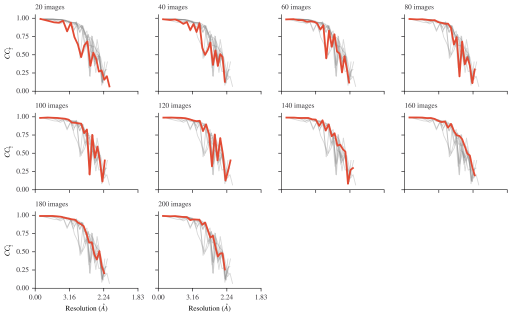

(e)

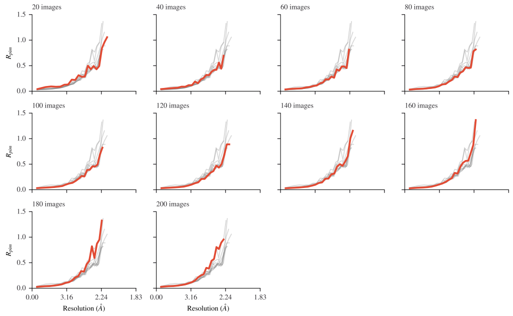

(f)

Figure S3: Comparison of merging statistics using only the first 20, 40, ..., 200 images from each data set for SARS-CoV-2 main protease ligand soaks (a) and (b) Z1367324110, (c) and (d) Z31792168, (e) and (f) Z4439011520.

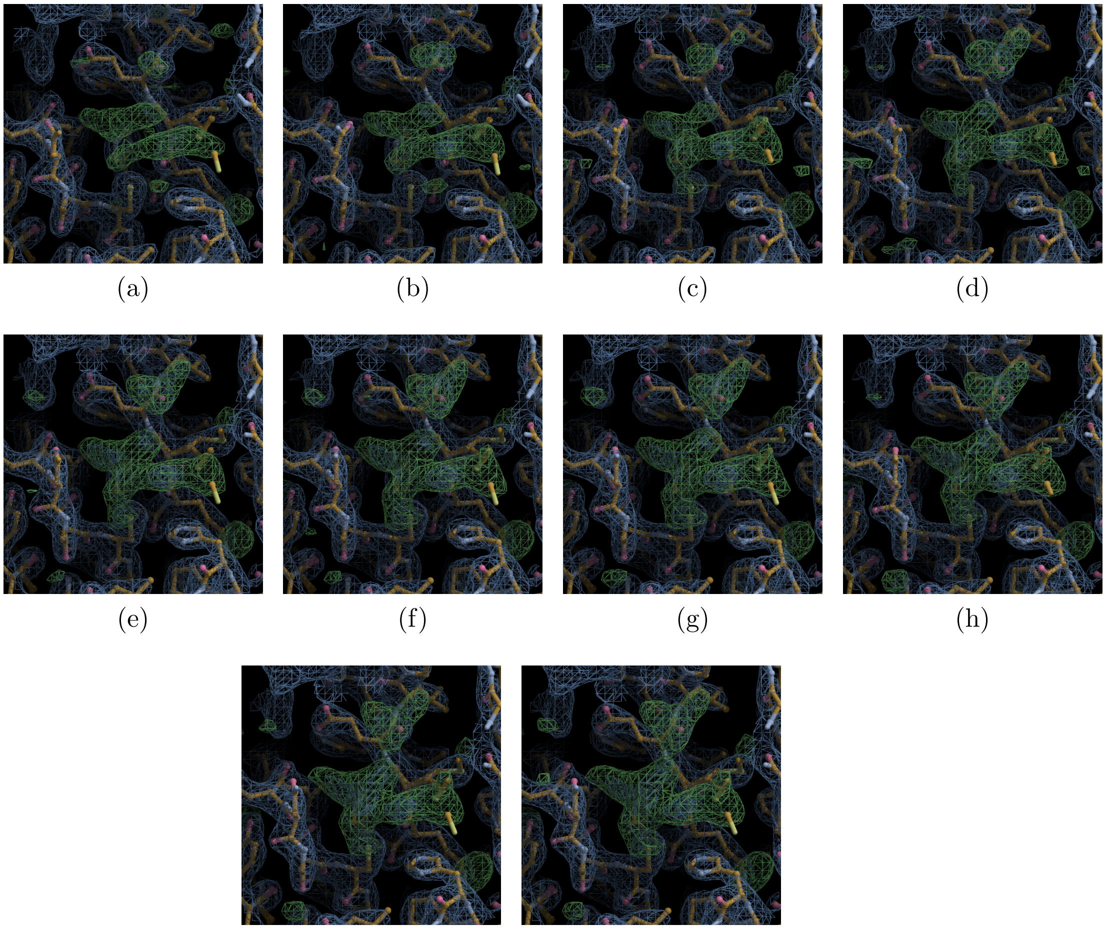

(a) (b) (c) (d)

(e) (f) (g) (h)

(i) (j)

Figure S4: *DIMPLE*-generated $2F_o - F_c$ (blue) and $F_o - F_c$ (green) electron density maps for SARS-CoV-2 main protease ligand soak Z4439011520 using only the first (a) 20, (b) 40, ..., (j) 200 images of each data set. All contours are drawn at 3σ.

Dose calculations

RADDOSE-3D input and output for dose calculation for a typical crystal as used in §5 hit side-on with the X-ray beam:

Input

```
##############################################################################
# Crystal Block #
##############################################################################

Crystal

Type Cuboid
# Crystal shape can be Cuboid or Spherical

Dimensions 5 50 50
# Dimensions of the crystal in X,Y,Z in µm.
# Z is the beam axis, Y the rotation axis and
# X completes the right handed set
# (vertical if starting face-on).

PixelsPerMicron 0.5
# This defines the coarseness of the simulation
# (i.e. how many voxels the crystal is divided into.)
# Preferably set as high as possible, however for a higher
# value the simulation will take longer to complete.
# Recommended to try increasing between 0.5 and 5 and ensure
# the reported dose value converges as PixelsPerMicron increases.
# As a rule of thumb, this needs to be at least 10x the beam
# FWHM for a Gaussian beam.
# e.g. 20 µm FWHM beam -> 2 µm voxels -> 0.5 voxels/µm

# NOTE: Use AngleP/AngleL if your crystal is not face-on to the beam.
# See RD3D user guide for more details

# Also need to specify the crystal composition below (Example case for insulin given):
AbsCoefCalc RD3D
# Absorption Coefficients calculated
# using RADDOSE-3D (Zeldin et al. 2013).

UnitCell 115.21 54.78 45.34 90 101.24 90
# unit cell size: a, b, c with alpha, beta and gamma angles default to 90°

NumMonomers 2
# number of monomers in unit cell

NumResidues 305
# number of residues per monomer

ProteinHeavyAtoms S 22
# heavy atoms added to protein part of the
# monomer, i.e. S, coordinated metals, Se in Se-Met

SolventHeavyConc S 700
```

```
# concentration of elements in the solvent
# in mmol/l. Oxygen and lighter elements
# should not be specified

SolventFraction 0.3716
# fraction of the unit cell occupied by solvent

##############################################################################
# Beam Block #
##############################################################################

Beam

Type Gaussian
# beam profile can be Gaussian or TopHat
# Flux 7e12
Flux 2.03e11
# in photons per second (2e12 = 2 * 10^12)
FWHM 30 30
# in µm, horizontal by vertical for a Gaussian beam
Energy 12.4
# photon energy in keV

Collimation Rectangular 100 100
# Horizontal/Vertical collimation of the beam
# For 'uncollimated' Gaussians, 3xFWHM recommended

##############################################################################
# Wedge Block #
##############################################################################

Wedge 0 20
# Start and End rotational angle of the crystal with Start < End

ExposureTime 2
# Total time for entire angular range

# AngularResolution 2
# Only change from the defaults when using very
# small wedges, e.g 5°.

# NOTE: To define more complex geometries (helical, de-centred, or offset),
# see the StartOffset, TranslatePerDegree, and RotAxBeamOffset keywords
# in the User Guide
```

Output

```
Cuboid (Polyhedron) crystal of size [5, 50, 50] um [x, y, z] at a resolution of 2.00 microns per voxel edge.
Simple DDM.
Gaussian beam, 100.0x100.0 um with 30.00 by 30.00 FWHM (x by y) and 2.0e+11 photons per second at 12.40 keV.
Wedge 1:
Collecting data for a total of 2.0s from phi = 0.0 to 20.0 deg.

Crystal coefficients calculated with RADDOSE-3D.
Photelectric Coefficient: 1.83e-04 /um.
Inelastic Coefficient: 1.29e-05 /um.
Elastic Coefficient: 1.36e-05 /um.
Attenuation Coefficient: 2.09e-04 /um.
Density: 0.78 g/ml.

Average Diffraction Weighted Dose         : 0.066779 MGy
Last Diffraction Weighted Dose            : 0.126371 MGy
Elastic Yield                             : 4.73e+07 photons
Diffraction Efficiency (Elastic Yield/DWD): 7.09e+08 photons/MGy
Average Dose (Whole Crystal)              : 0.108026 MGy
Average Dose (Exposed Region)             : 0.108026 MGy
Max Dose                                  : 0.183466 MGy
Average Dose (95.0 % of total absorbed energy threshold (0.05 MGy)): 0.121631 MGy
Dose Contrast (Max/Threshold Av.)         : 1.51
Used Volume                               : 100.0%
Absorbed Energy (this Wedge)              : 1.30e-06 J.
Dose Inefficiency (Max Dose/mJ Absorbed)  : 141.3 1/g
Dose Inefficiency PE (Max Dose/mJ Deposited): 144.4 1/g
Final Dose Histogram:
Bin  1,  0.0 to  0.1 MGy: 44.7 %
Bin  2,  0.1 to  3.4 MGy: 55.3 %
Bin  3,  3.4 to  6.7 MGy:  0.0 %
Bin  4,  6.7 to 10.1 MGy:  0.0 %
Bin  5, 10.1 to 13.4 MGy:  0.0 %
Bin  6, 13.4 to 16.7 MGy:  0.0 %
Bin  7, 16.7 to 20.0 MGy:  0.0 %
Bin  8, 20.0 to 23.4 MGy:  0.0 %
Bin  9, 23.4 to 26.7 MGy:  0.0 %
Bin 10, 26.7 to 30.0 MGy:  0.0 %
Bin 11, 30.0 MGy upwards:  0.0 %
```

RADDOSE-3D input and output for dose calculation for a typical crystal as used in §5 hit face-on with the X-ray beam:

Input

```
##############################################################################
# Crystal Block #
##############################################################################

Crystal

Type Cuboid
# Crystal shape can be Cuboid or Spherical

Dimensions 50 50 5
# Dimensions of the crystal in X,Y,Z in µm.
# Z is the beam axis, Y the rotation axis and
# X completes the right handed set
# (vertical if starting face-on).

PixelsPerMicron 0.5
# This defines the coarseness of the simulation
# (i.e. how many voxels the crystal is divided into.)
# Preferably set as high as possible, however for a higher
# value the simulation will take longer to complete.
# Recommended to try increasing between 0.5 and 5 and ensure
# the reported dose value converges as PixelsPerMicron increases.
# As a rule of thumb, this needs to be at least 10x the beam
# FWHM for a Gaussian beam.
# e.g. 20 µm FWHM beam -> 2 µm voxels -> 0.5 voxels/µm

# NOTE: Use AngleP/AngleL if your crystal is not face-on to the beam.
# See RD3D user guide for more details

# Also need to specify the crystal composition below (Example case for insulin given):
AbsCoefCalc RD3D
# Absorption Coefficients calculated
# using RADDOSE-3D (Zeldin et al. 2013).

UnitCell 115.21 54.78 45.34 90 101.24 90
# unit cell size: a, b, c with alpha, beta and gamma angles default to 90°

NumMonomers 2
# number of monomers in unit cell

NumResidues 305
# number of residues per monomer

ProteinHeavyAtoms S 22
# heavy atoms added to protein part of the
# monomer, i.e. S, coordinated metals, Se in Se-Met

SolventHeavyConc S 700
# concentration of elements in the solvent
# in mmol/l. Oxygen and lighter elements
```

```
# should not be specified

SolventFraction 0.3716
# fraction of the unit cell occupied by solvent

##############################################################################
# Beam Block #
##############################################################################

Beam

Type Gaussian
# beam profile can be Gaussian or TopHat
# Flux 7e12
Flux 2.03e11
# in photons per second (2e12 = 2 * 10^12)
FWHM 30 30
# in μm, horizontal by vertical for a Gaussian beam
Energy 12.4
# photon energy in keV

Collimation Rectangular 100 100
# Horizontal/Vertical collimation of the beam
# For 'uncollimated' Gaussians, 3xFWHM recommended

##############################################################################
# Wedge Block #
##############################################################################

Wedge 0 20
# Start and End rotational angle of the crystal with Start < End

ExposureTime 2
# Total time for entire angular range

# AngularResolution 2
# Only change from the defaults when using very
# small wedges, e.g 5°.

# NOTE: To define more complex geometries (helical, de-centred, or offset),
# see the StartOffset, TranslatePerDegree, and RotAxBeamOffset keywords
# in the User Guide
```

Output

```
Cuboid (Polyhedron) crystal of size [50, 50, 5] um [x, y, z] at a resolution of 2.00 microns per voxel edge.
Simple DDM.
Gaussian beam, 100.0x100.0 um with 30.00 by 30.00 FWHM (x by y) and 2.0e+11 photons per second at 12.40 keV.
Wedge 1:
Collecting data for a total of 2.0s from phi = 0.0 to 20.0 deg.

Crystal coefficients calculated with RADDOSE-3D.
Photelectric Coefficient: 1.83e-04 /um.
Inelastic Coefficient: 1.29e-05 /um.
Elastic Coefficient: 1.36e-05 /um.
Attenuation Coefficient: 2.09e-04 /um.
Density: 0.78 g/ml.

Average Diffraction Weighted Dose         : 0.050366 MGy
Last Diffraction Weighted Dose            : 0.095817 MGy
Elastic Yield                             : 2.02e+07 photons
Diffraction Efficiency (Elastic Yield/DWD): 4.01e+08 photons/MGy
Average Dose (Whole Crystal)              : 0.069056 MGy
Average Dose (Exposed Region)             : 0.069056 MGy
Max Dose                                  : 0.183705 MGy
Average Dose (95.0 % of total absorbed energy threshold (0.03 MGy)): 0.082169 MGy
Dose Contrast (Max/Threshold Av.)         : 2.24
Used Volume                               : 100.0%
Absorbed Energy (this Wedge)              : 5.53e-07 J.
Dose Inefficiency (Max Dose/mJ Absorbed)  : 332.0 1/g
Dose Inefficiency PE (Max Dose/mJ Deposited): 339.2 1/g
Final Dose Histogram:
Bin  1,  0.0 to  0.1 MGy: 74.2 %
Bin  2,  0.1 to  3.4 MGy: 25.8 %
Bin  3,  3.4 to  6.7 MGy:  0.0 %
Bin  4,  6.7 to 10.1 MGy:  0.0 %
Bin  5, 10.1 to 13.4 MGy:  0.0 %
Bin  6, 13.4 to 16.7 MGy:  0.0 %
Bin  7, 16.7 to 20.0 MGy:  0.0 %
Bin  8, 20.0 to 23.4 MGy:  0.0 %
Bin  9, 23.4 to 26.7 MGy:  0.0 %
Bin 10, 26.7 to 30.0 MGy:  0.0 %
Bin 11, 30.0 MGy upwards:  0.0 %
```
